# The Antibody Response to the BNT162b2 mRNA COVID-19 Booster in Healthcare Workers: Association between the IgG Antibody Titers and Anthropometric and Body Composition Parameters

**DOI:** 10.3390/vaccines10101638

**Published:** 2022-09-29

**Authors:** Marlena Golec, Adam Konka, Martyna Fronczek, Joanna Zembala-John, Martyna Chrapiec, Karolina Wystyrk, Sławomir Kasperczyk, Zenon Brzoza, Rafał Jakub Bułdak

**Affiliations:** 1 Silesian Park of Medical Technology Kardio-Med Silesia, M. Curie-Skłodowskiej 10C, 41-800 Zabrze, Poland; 2 Department of Pharmacology, Faculty of Medical Sciences in Zabrze, Medical University of Silesia in Katowice, H. Jordana 38, 41-808 Zabrze, Poland; 3 Department of Medicine and Environmental Epidemiology, Faculty of Medical Sciences in Zabrze, Medical University of Silesia in Katowice, H. Jordana 19, 41-808 Zabrze, Poland; 4 Silesian Center for Heart Diseases in Zabrze, M. Curie-Skłodowskiej 9, 41-800 Zabrze, Poland; 5 Department of Biochemistry, Faculty of Medical Sciences in Zabrze, Medical University of Silesia in Katowice, H. Jordana 19, 41-808 Zabrze, Poland; 6 Department of Internal Diseases, Allergology, Endocrinology and Gastroenterology, Institute of Medical Sciences, University of Opole, Al. W. Witosa 26, 40-451 Opole, Poland; 7 Department of Clinical Biochemistry and Laboratory Diagnostics, Institute of Medical Sciences, University of Opole, Oleska 48, 45-052 Opole, Poland

**Keywords:** IgG antibody, SARS-CoV-2, COVID-19, healthcare workers, humoral response, COVID-19 vaccine, BNT162b2 vaccine, immunization, body composition, booster

## Abstract

Background: Research shows that in most people, two-dose vaccination helps to shape the humoral response to Severe Acute Respiratory Syndrome Coronavirus 2 (SARS-CoV-2). Further studies are required to learn about the vaccine’s effectiveness after boosting. Methods: We conducted a prospective study among 103 healthcare workers (HCWs) from a regional multi-specialty hospital vaccinated with three doses of the BNT162b2 vaccine. We compared their immunoglobulin G (IgG) titers 14 days after the second dose with those 21 days after the booster. We also compared their anthropometric and body composition parameters with IgG concentrations at the same time points. Results: Twenty-one days after the booster, all study participants were seropositive. Their mean IgG antibody titers were significantly lower than 14 days after the second dose (158.94 AU/mL ± 90.34 AU/mL vs. 505.79 AU/mL ± 367.16 AU/mL). Post-booster Spearman’s correlation analysis showed a significantly weak correlation between the IgG antibody titer and parameters related to muscle tissue and adipose tissue (including body fat mass). Conclusions: The BNT162b2 booster stimulates the humoral response to a lesser extent than the two-dose BNT162b2 primary vaccination. The adipose and muscle tissue parameters show a weak positive correlation with the SARS-CoV-2 IgG antibody titers.

## 1. Introduction

Vaccination is still considered the most optimal protective measure against infection caused by Severe Acute Respiratory Syndrome Coronavirus 2 (SARS-CoV-2). Moreover, it is still considered the best strategy for controlling and combating the Coronavirus Disease 2019 (COVID-19) pandemic. Since the beginning, high hopes for overcoming this tremendous global public health crisis have been placed on developing vaccines and mass vaccination of the world’s population, thus achieving herd immunity [1,2]. Today, over 1.5 years after introducing the first anti-COVID-19 vaccines, this goal is still ahead of us, despite the broad rollout of vaccines in many countries. Although, according to recent estimations of August 9, 2022, the share of individuals who completed the initial vaccination course reached 61.88% of the global population, it varies significantly from one country to another, ranging from 88.67% in Cuba to 11.56% in Nigeria [3]. In the face of evolving SARS-CoV-2 and emerging variants of concern (i.a., Omicron’s subvariants), today the world concentrates on a more realistic goal: to minimize severe COVID-19 outcomes, including death, and reduce the overall burden related to the pandemic [2].

Apart from immunization, a key role in achieving that aim plays a better understanding of the factors affecting the magnitude and duration of the immune response to SARS-CoV-2. After the success of the first COVID-19 vaccines, including the BNT162b2 vaccine (Comirnaty; BioNTech/Pfizer, New York, NY, USA), measured by a noticeably reduced number of infections, hospitalizations and deaths, the next question was whether the immunity to SARS-CoV-2, achieved through two doses of vaccine, would be long-lasting and sufficient to protect from re-infection [4,5]. In addition, the rapid emergence of new coronavirus variants and subvariants of concern and differences in their reactions to SARS-CoV-2 specific antibodies have forced the need to investigate the immune response to vaccination [6].

With growing evidence of waning immunity over time, many countries have started administering a booster dose of vaccine to enhance and prolong the immune response [7]. The approach has a particular meaning in the case of healthcare workers (HCWs)—A professional group at higher risk of contracting COVID-19 due to its close contact with SARS-CoV-2 positive patients [8]. It is important to highlight that, according to the US Food and Drug Administration (FDA), SARS-CoV-2 antibody tests should not be used to assess an individual’s protection level against COVID-19 [9]. Cohort studies show an 80.00%–90.00% decrease in SARS-CoV-2 infection rates in antibody-positive people for at least 6 months after recovering from COVID-19. However, it is still unclear to what extent and for how long persons with detectable antibodies are protected against reinfection with SARS-CoV-2 or what concentration of antibodies is needed to provide such protection [10,11,12]. In order to determine whether antibody responses below a particular threshold are related to vaccine failure, including reinfection, longitudinal patient follow-up studies are being conducted to evaluate antibody levels before and after infection or vaccination. 

Predicting the course of COVID-19 and creating an effective pandemic control strategy depends on the interpretation of the humoral response and on understanding the basic features and evolution of B and T cell-mediated adaptive immunity to SARS-CoV-2 [13]. Research indicates that the cellular response is strongly developed after infection and vaccination, in parallel with the humoral response. However, it should be highlighted that the measurement of antibodies is the most frequently used method for assessing the body’s response to active immunization. There is increasing evidence that many SARS-CoV-2 positive subjects develop strong and long-lasting T-cell immunity, which is the basis of the cellular response [14]. Moreover, it should be noted that, in some people, the measurement of cellular response may be the main and only criterion for assessing cellular immunity following SARS-CoV-2 infection or vaccination, as they do not produce sufficient antibody levels to determine a humoral response. In such individuals, the immune response after vaccination can only be confirmed by measuring cellular immunity [15].

The aim of this study was to assess whether the intake of the third homologous dose of the BNT162b2 vaccine stimulates an early humoral response in the form of anti-SARS-CoV-2 immunoglobulin G (IgG) production in HCWs. An additional aim of the study was to verify whether the collected anthropometric and body composition parameters are correlated to the anti-SARS-CoV-2 IgG titers after the booster dose.

## 2. Materials and Methods

### 2.1. Study Group 

The research was conducted among the HCWs of the Regional Specialized Hospital no. 4 in Bytom (Upper Silesia, Poland), vaccinated with two doses of the BNT162b2 vaccine (homologous primary course), followed by a homologous BNT162b2 booster. Participants’ demographic parameters (sex, age, job position), primary clinical data (i.a., self-reported presence of chronic diseases), and COVID-19 history: i.a., no history of infection/being COVID-19 convalescent/unknown, date and type of SARS-CoV-2 test performed (real-time reverse transcription-polymerase chain reaction (RT-PCR) or SARS-CoV-2 antigen test), infection course (symptomatic/asymptomatic) and ongoing COVID-19 infection in the household before the study start were collected through an author questionnaire.

### 2.2. Study Design

The concept of this study was based on the pilot research conducted by the authors, aiming to investigate the humoral response of 243 HCWs on the timeline and factors affecting IgG titers during and after administration of a two-dose regimen of BNT162b2 vaccine [16]. 

In our research, we followed the national regulations and guidelines on vaccination strategies in this professional group. According to these official indications, the primary vaccination course for HCWs was obligatory, and a booster dose was highly recommended. For this group, the administration of the BNT162b2 vaccine was decided.

Vaccination and sera collection, as well as anthropometric and body composition measurements, were performed in the Regional Specialized Hospital no. 4 in Bytom by researchers from the Silesian Park of Medical Technology Kardio-Med Silesia (KMS). Moreover, the data and specimens were analyzed in the accredited COVID-19 research laboratory of KMS (Accreditation Certificate no. AB 1802, issued by the Polish Center for Accreditation). The study obtained the approval of the Institutional Review Board of the Medical University of Silesia in Katowice (PCN/0022/KB1/50/20) and was performed in accordance with the Helsinki Declaration.

All participants signed the informed consent forms prior to enrollment in the study. The inclusion criteria were: age above 18 years, signed informed consent to participate in the study, willingness to vaccinate with three doses of BNT162b2 vaccine in KMS, approval to undergo five blood draws in KMS following the study protocol, and finally no contraindications to vaccination and body composition measurements. The exclusion criteria were withdrawal of informed consent during the study period for personal or other reasons and deviation from the study protocol (i.a., not being able to participate in the blood collection according to the predicted blood collection schedule).

The primary vaccination course was conducted according to the World Health Organization Strategic Advisory Group of Experts on Immunization (WHO SAGE) interim recommendations of 8 January 2021, and the national immunization schedule for the priority-use group, i.e., healthcare professionals obliging at that time [17,18,19]. The first doses in this group were administered between 29 December 2020, and 15 January 2021; the second was between January 20 and 11 February 2021. A booster dose, following the national vaccination recommendations, was given in a period between 29 September to 21 October 2021 (a homologous schedule).

**Figure 1:**
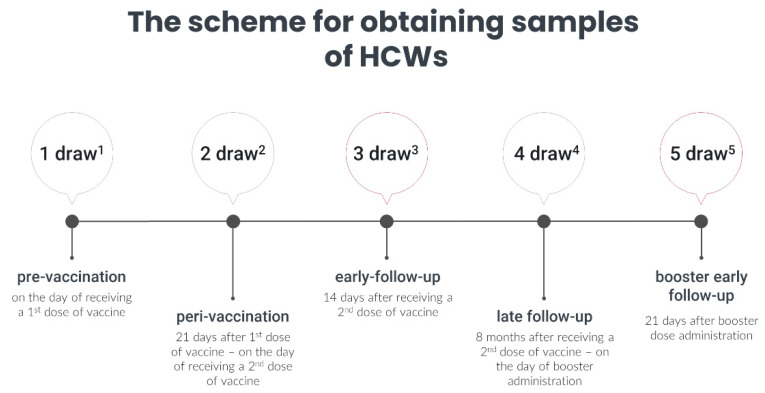
Schematic timetable of blood collections in the group of HCWs. ^1^ first draw before starting the vaccination; ^2^ second draw on the day of receiving the second dose of the BNT162b2 vaccine; ^3^ third draw 14 days after receiving the second dose of the BNT162b2 vaccine; ^4^ fourth draw 8 months after receiving the second dose of the BNT162b2 vaccine; ^5^ fifth draw 21 days after receiving the booster dose of the BNT162b2 vaccine.

The study protocol was divided into three parts—three stages: first, during the primary vaccination course, two blood collections were conduction; second, during the follow-up, there were two post-vaccination draws; and third, after receiving a booster, there was one blood draw (Figure 1). It is worth highlighting that during the study, three different variants of concern were dominant in the Upper Silesia region, i.e., the first Alpha (B.1.1.7), followed by Delta (B1.617.2), and finally, Omicron (B.1.1.529). The latter dominated during booster administration and the follow-up period [20].

### 2.3. Anti-SARS-CoV-2 IgG Measurement

Serological tests were performed using a two-step enzyme IgG chemiluminescent immunoassay—Access SARS-CoV-2 IgG II (Beckman Coulter Inc., Brea, CA, USA). This method is based on the patient’s serum reaction with a recombinant SARS-CoV-2 spike 1 protein (S1) containing the sequence of the receptor-binding domain (RBD). The results were defined according to the manufacturer’s instructions as reactive for SARS-CoV-2 (seropositive) when the titer of IgG against RBD of the S1 protein was ≥10.00 AU/mL and as non-reactive (seronegative) when the value of antibodies was <10.00 AU/mL. Our pilot study described the research methodology in detail [16].

### 2.4. Body Mass Index and Body Composition Measurement

The body mass index (BMI) was calculated based on body mass and height. The results were interpreted according to the WHO classification: <18.50 is underweight, 18.50–24.99—normal weight, 25.00–29.99—overweight, and ≥30.00—obesity [21]. In addition, waist circumference (WC) and hip circumference (HC) were measured; based on those variables, the waist-to-hip ratio (WHR) was calculated. 

Body composition was analyzed using a non-invasive TANITA Body Composition Analyzer MC-780MA (TANITA Corporation, Tokyo, Japan). Based on an electrical bioimpedance analysis (BIA), this device is certified and approved for clinical use with NAWI CLASS III standards for scales used for medical measurements (EU certification CE0122). It also complies with the Medical Device Directive (MDD 93/42/EEC). 

Before the examination, individuals’ data (identification number, sex, date of birth, body height, and clothing weight) were entered into the GMON program (GMON Pro 3.4.5, Medizin & Service GmbH, Chemnitz, Germany).

The research methodology using TANITA was described in detail in our previous paper [16]. The measurements were conducted following the manufacturer’s instructions and by the same study team members.

### 2.5. Statistical Analysis

Data were presented as mean ± standard deviation (SD) for variables with normal distribution and as median with interquartile range for variables with non-normal distribution. Data distribution was assessed using the Shapiro–Wilk test. Spearman’s correlation coefficient was used due to the non-normal distributions of parameters to assess the relationships between the examined variables. Friedman’s test was performed to compare multiple repeated measurements of the antibody concentrations. *p* values < 0.05 were considered statistically significant. The statistical analysis was performed using RStudio software (RStudio, PBC, Boston, MA, USA [22]). 

## 3. Results

In our study, 103 HCWs who received three doses of the BNT162b2 vaccine and met the inclusion criteria were enrolled: 79 women (76.70%) and 24 men (23.30%). The mean age in this group was 48.94 years (range: 25–86 years). The study group’s basic demographic and clinical characteristics are presented below (Table 1).

At baseline, 30 individuals (29.13%) were seropositive (≥10.00 AU/mL). Twenty-two participants (21.36%) in the study group underwent COVID-19, confirmed objectively by the test: 17 only by RT-PCR, 2 both by RT-PCR and antigen test, and 3 by antigen test exclusively. Interestingly, in this group, 6 HCWs (27.27%) were seronegative (<10.00 AU/mL) before receiving the first dose of vaccine, with a mean IgG titer of 2.03 AU/mL (range: 0.23–7.68 AU/mL). Among convalescents, only 9 (40.91%) were symptomatic. Out of 13 (59.09%) asymptomatic subjects, 6 confirmed having ongoing infection in their households at the time of their COVID-19 diagnosis. 

Another two HCWs declared passing COVID-19-like infection (not test-confirmed) before starting the primary vaccination course; neither reported ongoing infection in their households during symptom onset.

Fourteen days after receiving the second dose of vaccine, 101 subjects (98.06%) were seropositive. Eight months after completing the primary vaccination course, only 52 participants (50.49%) maintained a reactive level of anti-SARS-CoV-2 IgG titers (mean 34.77 AU/mL ± 32.71 AU/mL). However, IgG concentrations in this cohort significantly decreased compared to the previous measurement, regardless of the subjects’ COVID-19 history (being naïve or convalescent). 

Following the national vaccination policy guidelines, a booster dose was administered to extend protection against COVID-19 in this high-risk professional group. Undertaken action has proven effective: 21 days after boosting seropositivity was confirmed in all study participants (100.00%), but their mean IgG antibody titers were significantly lower than 14 days after the second dose (158.94 AU/mL ± 90.34 AU/mL vs. 505.79 AU/mL ± 367.16 AU/mL) (Table 2).

**Figure 2:**
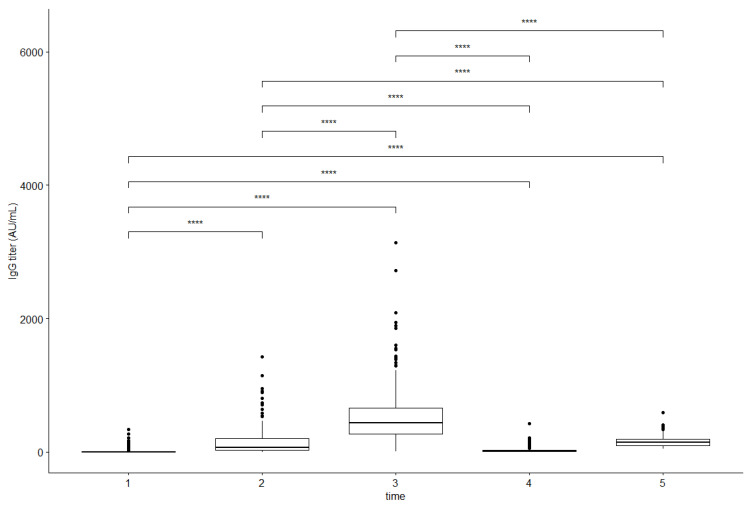
Changes in the anti-SARS-CoV-2 IgG titers over time: before the first dose (1), on the day of receiving a second dose, before the shot (2), 14 days after receiving the second dose (an early follow-up) (3), 8 months after complete inoculation (a long-term follow-up), before receiving a booster injection (4), and 21 days after receiving a booster dose (5). Abbreviations: ****—*p* < 0.001 for paired multiple comparison tests.

A comparison analysis revealed that the highest anti-SARS-CoV-2 antibody titers (496.11 AU/mL ± 371.84 AU/mL) were reported after vaccination with two doses of the BNTb162b vaccine in the primary vaccination course (Figure 2).

The second part of our study aimed to investigate a potential correlation between anthropometric measurements and body composition parameters and IgG antibody titers following booster administration.

*The correlation between post-booster anti-SARS-CoV-2 IgG antibody titer and basic anthropometric measurements and derived ratios, and body composition parameters*

**Figure 3:**
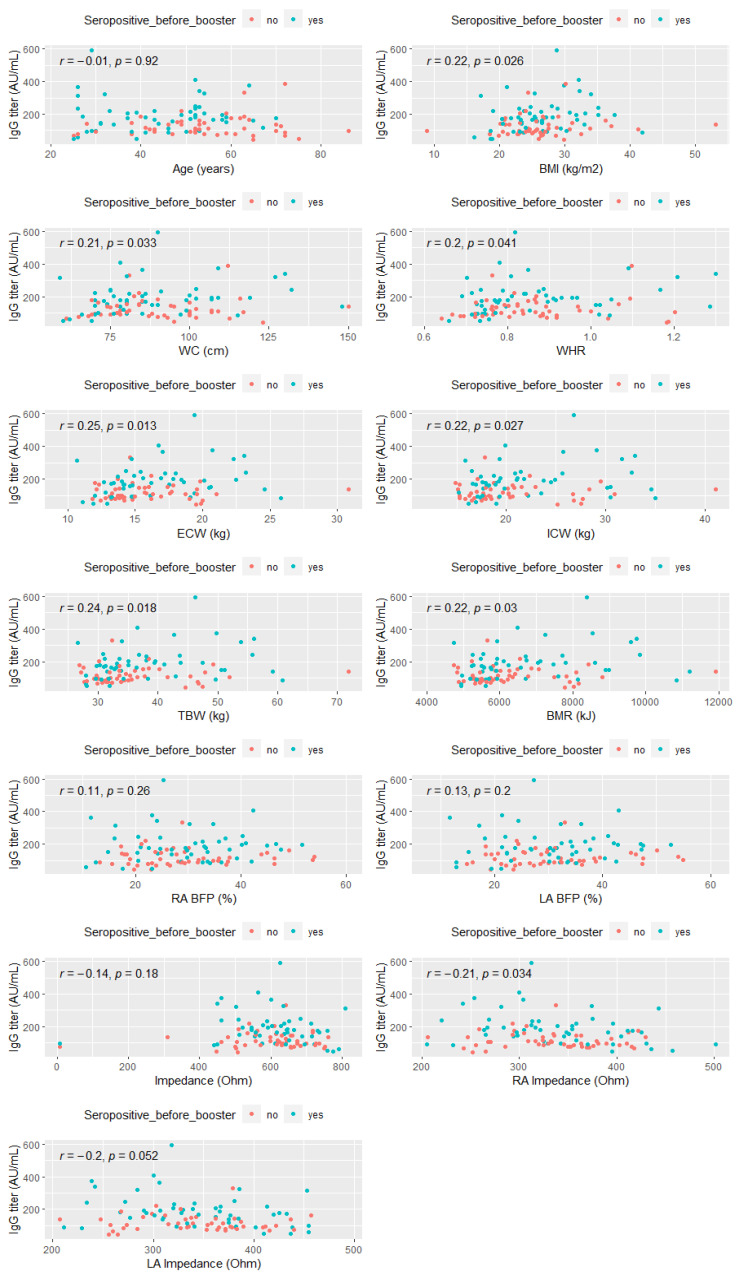
Spearman correlation between anti-SARS-CoV-2 IgG titers and selected anthropometric parameters and derived ratios and body composition parameters. Abbreviations: BMI—body mass index, WC—waist circumference, WHR—waist-hip ratio, TBW—total body water, ICW—intracellular water, ECW—extracellular water, BMR—basal metabolic rate, RA Impedance—right arm impedance, LA Impedance—left arm impedance.

Spearman’s correlation analysis, performed 21 days after the booster, revealed a significant weak relationship between anti-SARS-CoV-2 IgG antibody titers and the following basic anthropometric measurements and derived ratios: BMI (r = 0.22; *p* < 0.05), WC (r = 0.21; *p* < 0.05), WHR (r = 0.20; *p* < 0.05), total body water (TBW) (r = 0.24; *p* < 0.05), extracellular water (ECW) (r = 0.25; *p* < 0.05), intracellular water (ICW) (r = 0.22; *p* < 0.05), basal metabolic rate (BMR) (r = 0.22; *p* < 0.05). Moreover, a statistically significant weak negative correlation was observed between the anti-SARS-CoV-2 IgG antibody titers and right and left arm impedance (r = −0.21; *p* < 0.05 and r = −0.20; *p* < 0.05, respectively) (Figure 3).

*The correlation between post-booster anti-SARS-CoV-2 IgG antibody titer and selected parameters associated with the musculoskeletal system in the study group*

**Figure 4:**
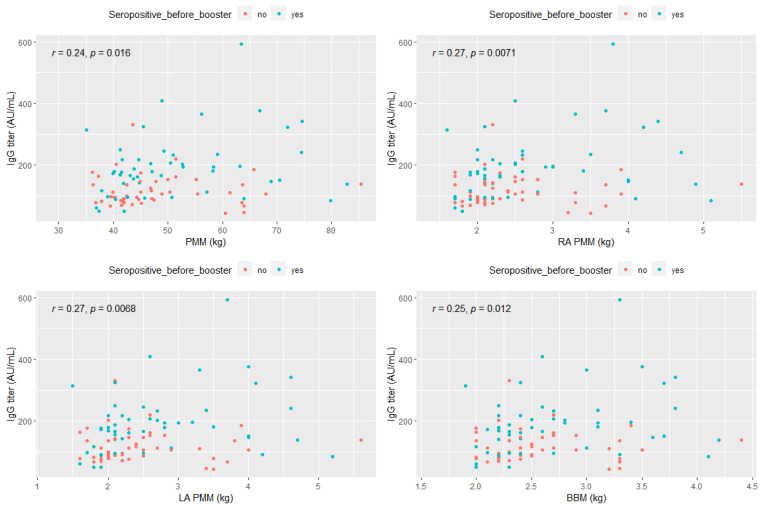
Spearman correlation between anti-SARS-CoV-2 IgG titer and selected parameters associated with the musculoskeletal system. Abbreviations: PMM—predicted muscle mass, RA PMM—right arm predicted muscle mass, LA PMM—left arm predicted muscle mass, BBM—body bone mass.

Spearman’s correlation analysis demonstrated a statistically significant weak positive correlation between anti-SARS-CoV-2 IgG titers and selected anthropometric parameters: predicted muscle mass (PMM) (r = 0.24, *p* < 0.05), right arm predicted muscle mass (RA PMM) (r = 0.27; *p* < 0.05), left arm predicted muscle mass (LA PMM) (r = 0.27; *p* < 0.05), and body bone mass (BBM) (0.25; *p* < 0.05) (Figure 4).

*The correlation between post-booster anti-SARS-CoV-2 IgG antibody titer and adipose tissue levels in the study group*

**Figure 5:**
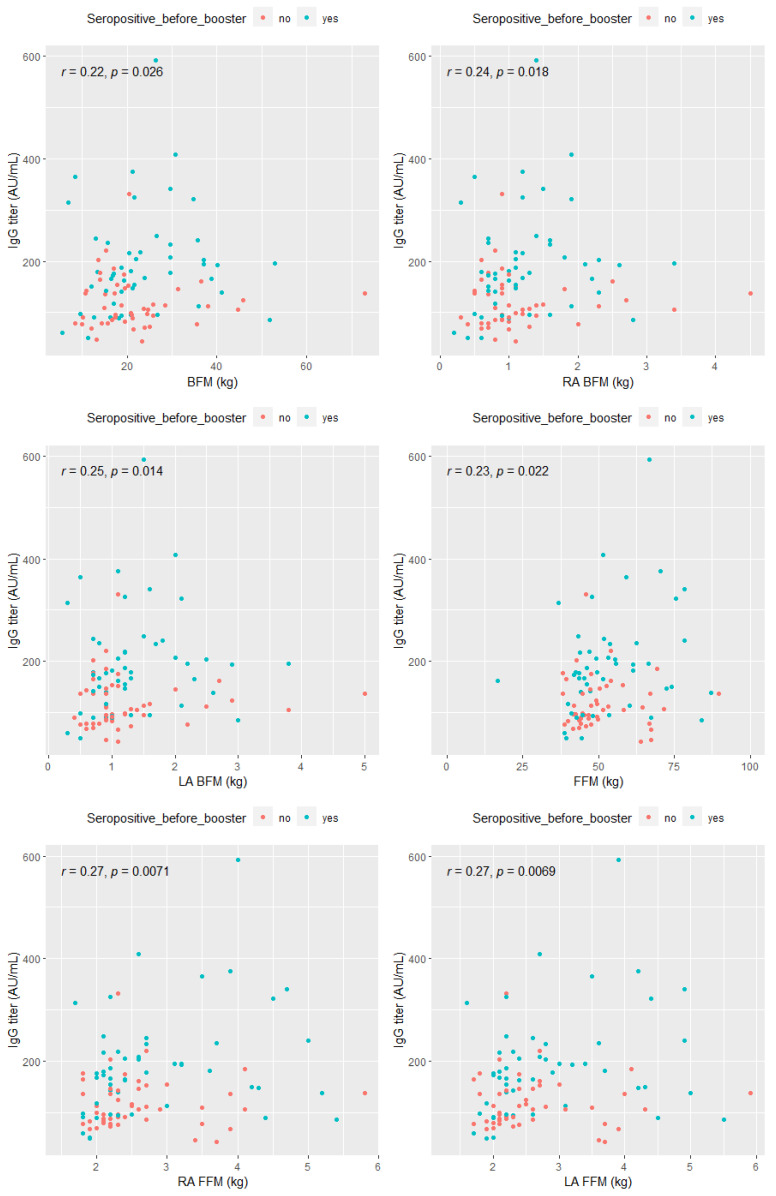
Spearman correlation between anti-SARS-CoV-2 IgG titers and selected parameters associated with adipose tissue level. Abbreviations: BFM—body fat mass, RA BFM—right arm body fat mass, LA BFM—left arm body fat mass, FFM—fat-free mass, RA FFM—right arm fat-free mass, LA FFM—left arm fat-free mass.

In addition, subsequent analysis using Spearman’s correlation method indicated a statistically significant weak positive correlation between post-booster anti-SARS-CoV-2 IgG antibody titers and the following adipose tissue-related parameters: body fat mass (BFM) (r = 0.22; *p* < 0.05), right arm body fat mass (RA BFM) (r = 0.24; *p* < 0.05), left arm body fat mass (LA BFM) (r = 0.25; *p* < 0.05), fat-free mass (FFM) (r = 0.23; *p* < 0.05), right arm fat-free mass (RA FFM) (r = 0.27; *p* < 0.05), left arm fat-free mass (LA FFM) (r = 0.27; *p* < 0.05) (Figure 5).

## 4. Discussion

Since the beginning of the COVID-19 pandemic, data on the persistence time of plasma cells and their ability to produce antibodies against SARS-CoV-2 have awakened great interest in the medical and scientific communities. Introducing the first vaccines to the real world has made it clear that a better understanding of antibody dynamics following COVID-19 vaccination is essential for designing effective, long-term vaccination strategies.

Despite an intensive search, a correlation of protection (CoP) against SARS-CoV-2 has not yet been determined [23]. Moreover, current studies on that topic have resulted in mixed findings, which can partially be explained by methodological differences, different measurement times and study protocols applied. However, a growing body of evidence indicates that the presence of IgG antibodies against the S1 protein could be associated with a reduced risk of SARS-CoV-2 infection or reinfection [11,23,24].

The immunoassay detecting binding antibody titer used in our work is widely available in most laboratories and is easy to apply. They also allow for a large-scale assessment of population antibody-mediated protection against COVID-19 following vaccination. Although the measurement of neutralizing antibodies (NAbs) appears to be the best approach to evaluate vaccine efficacy, some studies have shown a correlation between NAbs and efficacy and binding antibody titer and efficacy. Kung et al., for example, in their study, demonstrated a strong correlation between NAb titers (live SARS-CoV-2 neutralization assay) and anti-S1 binding IgG (r = 0.90) or anti-RBD IgG (r = 0.93) concentrations [25]. Gniadek et al., in their study using three different serology assays, discovered a positive, although of varying strength (r = 0.37–0.52) relationship between NAbs and antigen binding titers [26].

In our study, we concentrated on assessing anti-SARS-CoV-2 RBD domain IgG concentrations. We investigated the IgG antibody-mediated response to three doses of the BNT162b2 vaccine, focusing particularly on a booster-induced response. Interestingly, the most significant increase in anti-SARS-CoV-2 IgG antibody titers on the timeline was observed after administration of the second vaccine dose (early follow-up), as described previously [16]. At that measurement point, only two participants had non-reactive values of anti-SARS-CoV-2 IgG titers. Eight months later (late follow-up); however, before the booster administration, decreased IgG concentrations were reported in all individuals, and non-reactive subjects accounted for almost half of the study group. This pattern of anti-SARS-CoV-2 IgG kinetics and a similar duration of humoral response following the primary vaccination series was also observed in other serological studies conducted among HCWs [27,28]. Brisotto et al. reported a significant antibody decline four months after a two-dose primary vaccination [29]. 

To enhance immunity and restore protection against COVID-19, a booster dose was introduced. Following the national recommendation and vaccination schedule, approximately eight months after completing the primary vaccination course with the BNT162b2 vaccine, our study group received a homologous boost [30]. The booster again elucidated a humoral response, resulting in positive values of anti-SARS-CoV-2 IgG titers (≥10 AU/mL) in all study participants. However, the mean IgG antibody titer after the third dose was about 3-fold lower compared to the results obtained after administration of the second vaccine dose described in our previous study [16]. Contrary results were obtained by Skorupa et al., who reported significantly higher levels of anti-SARS-CoV-2 IgG antibodies after the BNT162b2 booster compared to those achieved after the second dose [31]. Nevertheless, again, the lack of standardized assessment methods, different time intervals between the primary two doses and a booster application, and finally, different measurement points of anti-SARS-CoV-2 IgG antibody concentrations make the results difficult to compare. 

Regardless of ambiguous findings on the post-booster magnitude of the humoral response, administration of a boosting dose seems to be a worthwhile strategy for combating COVID-19. Most studies confirmed an enhanced humoral response after boosting and observed an increase in seropositivity after a booster vaccination. Moreover, a study by Arbel et al. found that participants vaccinated with a two-dose regimen and booster had a 90% lower mortality rate than those who completed only a primary vaccination series [32]. A large study by Bar-On et al., conducted in a group of the elderly in Israel, reported that among participants who received a third vaccine dose, COVID-19 infection and its more severe course occurred significantly more seldom than among those vaccinated only with the primary vaccination course [11]. Finally, an interesting finding was reported by Gutmann et al., who discovered that the titer of anti-SARS-CoV-2 IgG-binding antibodies and neutralization capacity could be linked to circulating SARS-CoV-2 RNA detected in patients’ blood samples (RNAemia). In their study, RNAemia-positive patients had significantly lower anti-SARS-CoV-2 IgG antibodies and presented a lower neutralization capacity in the NAbs test. These observations shed new light on the use of antibody detection tests in combination with RNAemia determination as a prognostic marker for hospitalized COVID-19 patients [33].

In addition to monitoring the anti-SARS-CoV-2 IgG antibody titers on the timeline and comparing the results before and after the booster, our study investigated the correlation between the antibody-mediated response and selected anthropometric and body composition parameters, potentially affecting the body’s humoral response to the booster. 

Our previous study on a larger group of 243 HCWs presented the impact of adipose tissue and muscle tissue levels on immunization after the primary vaccination course with the BNT162b2 vaccine. We found that increased muscle and decreased fat mass can positively affect long-term immunity after vaccination, which is understood as the maintenance of higher anti-SARS-CoV-2 IgG titers [16]. This work, conducted on a smaller cohort of HCWs who received a booster dose aimed to investigate this phenomenon deeper, in a longer time perspective. 

In this study, we performed Spearman correlation analysis between the anti-SARS-CoV-2 IgG antibody titers and anthropometric parameters and derived ratios, such as BMI, WC, WHR and BMR, and body composition parameters, such as TBW, ECW and ICW. Twenty-one days after the booster administration, we observed a weak positive correlation between the above-mentioned variables and anti-SARS-CoV-2 IgG titers. These results contradict other studies on that topic in which such correlation was not reported. In the present research, booster-induced anti-SARS-CoV-2 IgG antibody titers increased with increasing anthropometric parameters. Interestingly, Watanabe et al. demonstrated that higher WC was associated with lower anti-SARS-CoV-2 IgG antibody titers but did not find a link between BMI and concentration of IgG antibodies after vaccination [34]. The lack of correlation between BMI and antibody titers after SARS-CoV-2 vaccination was also noticed by Parthymou et al. [35]. Likewise, Lustig et al. also did not report a significant influence of BMI on vaccine-induced anti-SARS-CoV-2 IgG antibody concentrations [36]. 

Nevertheless, data on a positive correlation between BMI and the magnitude of the humoral response are ambiguous, probably due to other factors affecting one’s antibody-mediated response (e.g., genetic and behavioral determinants, variant of SARS-CoV-2, etc.). Some studies, however, similar to ours, confirm a positive association between these variables [37]. It must be stressed, however, that apart from our research, all the above-mentioned studies refer to the values of anti-SARS-CoV-2 IgG titers obtained after administration of the second vaccine dose and not after the booster. Data on the association between the basic anthropometric parameters and body composition variables and the magnitude of the humoral response following boosting remain scarce and need further investigation.

It should also be highlighted that BMI is not the best indicator for determining body fat, as it has some significant limitations. First, it does not inform about the distribution of adipose tissue in different parts of the body; hence, it cannot be used to distinguish adipose tissue from lean tissue [38]. In addition, participants’ sex may skew the outcomes and result in incorrect BMI classification due to the different distribution of body fat in males and females. Nutall et al. pointed out that women generally have higher levels of BFM than men, yet their BMI is usually lower than that of men [38]. Therefore, research based solely on BMI and its association with adaptive immune response may be biased. In addition, this indicator can be used in large population studies and is not appropriate for small study groups. A more accurate tool for determining body fat is an electrical BIA [39,40]. 

Finally, we also performed the Spearman correlation analysis for two groups of variables: those related to the musculoskeletal system (PMM, RA PMM, LA PMM, BBM) and adipose tissue (BFM, RA BFM, LA BFM, RA FFM, LA FFM). After boosting, all analyses revealed weak positive correlations between the investigated parameters and the anti-SARS-CoV-2 IgG antibody titers. The analysis showed that PMM positively correlates with anti-SARS-CoV-2 IgG antibody concentrations, which is consistent with our previous study [16]. In addition, we found a weak positive correlation between BFM and anti-SARS-CoV-2 IgG titers. Similar findings were reported by mentioned earlier Watanabe et al., who observed lower COVID-19 mRNA vaccine-induced antibody titers in individuals with central obesity [34]. 

This study compared the anthropometric data and body composition parameters with anti-SARS-CoV-2 IgG antibody titers at different time intervals, i.e., three weeks following vaccine injection, and not eight months, as was performed in the pilot study by Golec et al. [16]. A significantly shorter follow-up period could contribute to the different correlation results between these studies. Longer time intervals were associated with decreased anti-SARS-CoV-2 IgG antibody concentrations. 

Skeletal muscles have been increasingly recognized as immune system regulators for several years. By disrupting homeostasis, a reduction in muscle mass may result in a reduced immune response [41]. Our study also observed this phenomenon, which showed a slight but statistically significant association between those variables: the anti-SARS-CoV-2 titers increased with increasing muscle tissue content. 

It has been confirmed that obese individuals respond poorly to infections [42,43,44], vaccination [45,46,47], and therapies [48]. Retrospective analyses of adult COVID-19 symptomatic patients have revealed that obese subjects (BMI ≥ 30) are more likely to be admitted to acute and critical care units compared to individuals with lower BMI values (BMI < 30) [49]. The adipose tissue in obese individuals is massively infiltrated with immune cells [50,51], causing local inflammation. Once activated after SARS-CoV-2 infection, the infiltrating immune cells contribute to the release of inflammatory mediators. In addition, adipose tissue, located in the thorax and abdominal areas, induces the secretion of additional pro-inflammatory mediators that can further compromise lung function [52,53]. It may become a viral reservoir, playing a crucial role in maintaining local and systemic inflammation and impairing immune function [54].

It must be stressed that anti-SARS-CoV-2 IgG antibodies cannot be considered the only definite marker of effective or failing adaptive immunity to SARS-CoV-2 following booster administration. The significance of, e.g., NAbs and cellular response to SARS-CoV-2 cannot be underestimated. Data on the contribution and role of each of these elements in providing sufficient protection against COVID-19 are evolving. In addition, circulating and emerging variants of the concerns of SARS-CoV-2 can also account for different responses of the body to contact with the pathogen [55,56]. 

In conclusion, in light of the above-mentioned findings, it seems crucial to monitor and evaluate vaccines’ effectiveness on the timeline and intensify the research on finding the firm, reliable correlate of protection against SARS-CoV-2.

Our study has a few limitations. First, the research was conducted on a relatively small test group. Second, passing COVID-19 infection was reported by the study participants exclusively through personal questionnaires and not verified objectively, which could result in overlooking asymptomatic or not-tested employees. Third, our study group was strongly feminized (a characteristic feature of this professional group in many countries), which could also translate into interpreting the results, especially those related to anthropometry and body composition.

Perspectives: SARS-CoV-2 is dynamically evolving. Despite a growing body of knowledge on the immune response to this pathogen, its novel variants continue to challenge us, raising new questions and concerns. Therefore, further and more extensive research is needed to better understand immune-building mechanisms following mRNA vaccine administration and the factors affecting the waning of vaccine-induced immunity, especially in high-risk groups, including obese individuals. 

In addition, it is worth considering more complex methods of post-vaccinal immunity assessment, including the measurements of viral load, especially in individuals with breakthrough infections. 

Such knowledge and abilities will help us better control and protect from COVID-19 and prepare for future pandemics.

## 5. Conclusions

The administration of the third dose of the BNT162b2 vaccine increases the concentration of IgG antibodies in the group of HCWs. Vaccination with a single injection booster significantly increases IgG antibody titers, as assessed by a short-term humoral response against SARS-CoV-2, but not as effectively as after the primary two-dose vaccination. In addition, the parameters related to adipose and muscle tissue show a weak positive correlation with anti-SARS-CoV-2 IgG antibodies following the booster. Additional statistical analyses and patients’ monitoring should be performed on a larger group of subjects to show whether the tested anthropometric parameters have an effect on the concentration of SARS-CoV-2 IgG titers after vaccination with the BNT162b2 booster and if there is a correlation between IgG titers and relative risk (RR) of severe disease or death. 

## Figures and Tables

**Table 1:** Basic demographic and clinical characteristics of the study group.

Parameters	*n* (%) 103 (100.00)	
Women	79 (76.70)	
Men	24 (23.30)	
Medical staff ^1^	81 (78.64)	
Auxiliary medical staff ^2^	14 (13.59)	
Administrative staff ^3^	8 (7.77)	
Chronic diseases ^4^	32 (31.07)	
Obesity (BMI ≥ 30.00)	22 (21.36)	
	**Mean ± SD**	**Median (Q1; Q3)**
Age (years)	48.98 ± 13.76	51.00 (39.00; 59.00)
Metabolic age (years)	44.37 ± 16.52	44.00 (33.00; 57.00)
BMI (kg/m^2^)	26.32 ± 5.90	25.56 (23.04; 29.05)
WC (cm)	88.55 ± 18.49	85.00 (75.00; 100.00)
HC (cm)	103.05 ± 11.66	102.00 (97.00; 110.00)
WHR	0.86 ± 0.14	0.82 (0.75; 0.92)
ECW (%)	16.18 ± 3.48	15.00 (13.70; 18.40)
ICW (%)	21.08 ± 5.30	19.20 (17.50; 23.50)
BBM (kg)	2.63 ± 0.56	2.40 (2.20; 3.00)
BFP (%)	29.14 ± 7.95	28.30 (24.00; 35.50)
BFM (kg)	22.49 ± 10.96	20.30 (15.40; 26.60)
FFM (kg)	52.05 ± 12.39	47.50 (43.80; 59.20)
TBW (%)	37.23 ± 8.62	34.00 (31.30; 41.60)
PMM (kg)	49.70 ± 11.33	45.10 (41.60; 56.20)
Impedance (Ohm)	602.65 ± 125.32	625.00 (546.00; 673.00)
BMR (kJ)	6524.55 ± 1495.80	5924.00 (5481.00; 7456.00)
VAT (level)	7.38 ± 5.04	7.00 (4.00; 10.00)
RA BFP (%)	29.58 ± 9.82	28.90 (22.50; 35.80)
RA BFM (kg)	1.20 ± 0.72	1.00 (0.70; 1.40)
RA FFM (kg)	2.72 ± 0.90	2.30 (2.10; 3.10)
RA PMM (kg)	2.58 ± 0.84	2.20 (2.00; 2.90)
RA Impedance (Ohm)	336.19 ± 59.51	335.00 (295.00; 376.00)
LA BFP (%)	30.67 ± 9.74	30.20 (23.50; 36.20)
LA BFM (kg)	1.29 ± 0.80	1.00 (0.80; 1.50)
LA FFM (kg)	2.74 ± 0.92	2.40 (2.10; 3.10)
LA PMM (kg)	2.60 ± 0.86	2.30 (2.00; 2.90)
LA Impedance (Ohm)	343.67 ± 60.09	341.00 (301.00; 382.00)

Legend: ^1^ Medical staff (doctors, nurses, paramedics); ^2^ Auxiliary medical staff-other healthcare professionals that were having direct contact with patients or medical specimens (i.a., physiotherapists, psychologists, technicians, diagnosticians, cleaners) ^3^ Administrative staff (medical secretaries, accountants), ^4^ Chronic diseases—cardiovascular diseases including arterial hypertension, diabetes, autoimmune diseases, asthma, allergy. Abbreviations: SD—standard deviation; Q1—first quartile; Q3—third quartile; BMI—body mass index; WC—waist circumference; HC—hip circumference; WHR—waist-hip ratio; ECW—extracellular water; ICW—intracellular water; BBM—body bone mass; BFP—body fat percentage; BFM—body fat mass; FFM—fat-free mass; TBW—total body water; PMM—predicted muscle mass; BMR—basal metabolic rate; VAT—visceral adipose tissue; RA—right arm; LA—left arm.

**Table 2:** Anti-SARS-CoV-2 IgG titer concentration in seronegative and seropositive participants at selected points of the time during the vaccination process with the BNT162b2 vaccine.

Vaccination Process	Anti-SARS-CoV-2 IgG Antibody Titer (AU/mL)					
	Seronegative (<10.00 AU/mL)			Seropositive (≥10.00 AU/mL)		
	*n* (%)	Mean ± SD	Median (Q1; Q3)	*n* (%)	Mean ± SD	Median (Q1; Q3)
**Before** **the first** **dose ^1^**	73 (70.87)	0.88 ± 2.05	0.21 (0.14; 0.35)	30 (29.13)	41.50 ± 48.50	24.90 (12.89; 39.91)
**Before** **the second** **dose ^2^**	13 (12.62)	5.52 ± 3.06	5.44 (3.73; 8.25)	90 (87.38)	153.53 ± 183.15	75.28 (33.16; 243.15)
**Early follow-** **up ^3^**	2 (1.94)	7.19 ± 0.68	7.19	101 (98.06)	505.79 ± 367.16	425.17 (267.85; 620.74)
**Late follow-** **up ^4^** **before** **the booster dose**	51 (49.51)	5.77 ± 2.12	5.84 (4.37; 7.27)	52 (50.49)	34.77 ± 32.71	22.15 (13.93; 42.86)
**Early follow-up** **after the** **booster ^5^**	0 (0.00)	-	-	103 (100.00)	158.94 ± 90.34	142.80 (94.03; 193.16)

Legend: ^1^ before the first dose-on the day of receiving a first dose of vaccine; ^2^ before the second dose-on the day of second dose administration; ^3^ early follow-up—14 days after completing a 2-dose regimen; ^4^ late follow-up—8 months after completing primary vaccination course, on the day of booster administration; ^5^ 21 days after booster administration. Abbreviations: *n* (%)—number of respondents (percentage of respondents); SD—standard deviation; Q1—first quartile; Q3—third quartile; IgG—immunoglobulin G; Q1—first quartile; Q3—third quartile; SD—standard deviation.

## Data Availability

The data used to support the findings of this research are available from the corresponding authors upon request.
